# Comparison of Tax-1 and Tax-2B post-translational modifications using specific lysine mutants in relation to activation of NF-κB and intracellular localization

**DOI:** 10.1186/1742-4690-8-S1-A143

**Published:** 2011-06-06

**Authors:** Marco Turci, Gianfranco Di Gennaro, Alessia Cotena, Oriano Marin, Francesca Avesani, Giorgia Cremonese, Erica Diani, Maria Romanelli, Umberto Bertazzoni

**Affiliations:** 1 Department of Life and Reproduction Sciences, Section of Biology and Genetics, Università degli Studi di Verona, Verona, 37134, Italy; 2 Department of Biological Chemistry, Università degli Studi di Padova , Padova, 35131, Italy

## 

Post-translational modifications of HTLV-1 and HTLV-2 Tax-1 and Tax-2 proteins have been shown to play a critical role in their cellular localization, transactivation and protein interactions. Five of ten lysine residues were found to be major targets for Tax-1 modifications: Lys189(K4); Lys197(K5), Lys263(K6), Lys280(K7) and Lys284(K8), are essential for ubiquitination, while sumoylation takes place on Lys280 (K7) and Lys284(K8). Tax-2 contains four additional lysine residues, namely at position Lys100(K2i), Lys149(K3i), Lys185(K3ii), and Lys356(K10i).

Very few studies have been so far performed on Tax-2 lysine mutants. We have previously demonstrated that Tax-2B is ubiquitinated and sumoylated similarly to Tax-1. To identify the Tax-2 lysine residues which are directly involved in post-translational modifications, we have constructed a series of Tax-2B mutants with substitutions of lysine (K) residues by arginines (R) and analyzed them for NF-kB and CREB/ATF transactivation, intracellular distribution and extent of ubiquitination and sumoylation. We have found that Tax-2 K7-8R mutant, contrary to its Tax-1 homologue, is only partially affected in its capacity to transactivate NF-κB pathway, is regularly sumoylated and presents formation of nuclear bodies by confocal analysis. However, Tax-2 mutants with extended (K3ii-8R) and/or total (K1-10iR) mutation rate were severely affected for NF-kB transactivation and sumoylation. By comparing Tax-2 WT with mutants K7-8R and K3ii-8R, we observed that the reduction of NF-κB activity is correlated to a parallel decrease in sumoylation. These results suggest that the target for Tax-2 ubiquitination and sumoylation differs from that described for Tax-1.

